# Evolution of treatment strategies for gastric cancer

**DOI:** 10.1002/ags3.12729

**Published:** 2023-08-10

**Authors:** Yoshihiro Kakeji

**Affiliations:** ^1^ Division of Gastrointestinal Surgery, Department of Surgery Kobe University Graduate School of Medicine Kobe Japan

With the decline in *Helicobacter pylori* infection rates, the incidence and mortality of gastric cancer have gradually decreased. However, gastric cancer remains the fifth most common cancer worldwide.^1^ Various clinical trials have led to updates in both surgical resection and systemic drug therapy as treatment strategies.

In this issue, Yanagimoto et al.^2^ present a comprehensive review of perioperative and surgical treatments for gastric and esophagogastric junction cancer (EGJC). Surgery remains the cornerstone of treatment for all stages of gastric cancer, with the standard gastrectomy involving resection of at least two‐thirds of the stomach with a D2 lymph node dissection. Recent large‐scale randomized controlled trials have shown that extended surgery does not significantly improve prognosis. Laparoscopic gastrectomy has proven feasible and effective for early to advanced stages, while ongoing trials are evaluating the efficacy of robotic surgery. EGJC is another important focus of treatment. The complex lymphatic drainage pathways have led to discussions on surgical approach and the extent of lymph node dissection for EGJC. A prospective nationwide multicenter study may provide insights into this clinical question. Additionally, clinical trials will compare triplet regimens, such as FLOT (fluorouracil plus leucovorin, oxaliplatin, and docetaxel) in Europe and DOS (docetaxel plus oxaliplatin and S‐1) in Asia, as perioperative chemotherapy for gastric cancer and EGJC. The combination of molecular‐targeted therapy or immunotherapy with chemotherapy shows promise in improving survival rates.

Improved outcomes for advanced gastric cancer are likely attributed to preoperative tumor burden reduction through immunochemotherapy, standardized surgical techniques for resection, and postoperative eradication of tumor cells. How about reduction surgery for gastric cancer? The REGATTA trial (JCOG0705/KGCA01) explored the survival benefits of reduction gastrectomy in patients with advanced gastric cancer and a single non‐curable factor but failed to demonstrate the superiority of gastrectomy plus chemotherapy over chemotherapy alone.^3^ Terashima et al.^4^ reported supplementary analysis aimed to explore the subgroups for which reduction gastrectomy might be beneficial with special reference to the tumor location and the country. The survival was slightly better in the gastrectomy plus chemotherapy arm than in the chemotherapy alone arm in the L‐lesion, especially in Korea. The number of chemotherapy treatment cycles appeared to be a significant factor contributing to improved survival after distal gastrectomy in Korea. Although primary chemotherapy remains the standard of care for advanced gastric cancer with non‐curable factors, the survival benefits of distal gastrectomy for reduction in tumors at the distal stomach remain controversial.

Following the eradication of *H. pylori‐*induced gastric cancer, the management of hereditary gastric cancer remains an important consideration. Iwatsuki et al.^5^ reviewed gastric adenocarcinoma and proximal polyposis of the stomach (GAPPS) from diagnosis to treatment. In cases of dysplasia, prophylactic total gastrectomy is recommended; however, due to its invasiveness and potential post‐gastrectomy syndrome, careful consideration of the patient's background is essential when making treatment decisions. The authors propose the establishment of a global registry system to systematically accumulate data on these rare cases in the future.

Robotic surgical systems with three‐dimensional camera and a high‐precision instrument operation will perform more accurate and curative procedures even in advanced stage with less complications. Newly developed agents will demonstrate robust anticancer efficacy. The integration of multimodal chemotherapy or immunotherapy with surgery holds the potential to further improve the prognosis of gastric cancer.

## FUNDING INFORMATION

This editorial did not receive any specific grant from funding agencies in the public, commercial, or nonprofit organizations.

## CONFLICT OF INTEREST STATEMENT

The author declares no conflict of interest for this article.

## ETHICS STATEMENT

Approval of the research protocol: Not Applicable.

Informed consent: Not Applicable.

Registry and the Registration No. of the study/Trial: Not Applicable.

Animal Studies: Not Applicable.
